# Perceptions of short-term medical volunteer work: a qualitative study in Guatemala

**DOI:** 10.1186/1744-8603-5-4

**Published:** 2009-02-26

**Authors:** Tyler Green, Heidi Green, Jean Scandlyn, Andrew Kestler

**Affiliations:** 1 School of Medicine, University of Colorado Denver, Denver, Colorado, USA; 2 Departments of Anthropology and Health and Behavioral Sciences, University of Colorado Denver, Denver, Colorado, USA; 3 Division of Emergency Medicine, University of Colorado Denver, Denver, Colorado, USA

## Abstract

**Background:**

Each year medical providers from wealthy countries participate in short-term medical volunteer work in resource-poor countries. Various authors have raised concern that such work has the potential to be harmful to recipient communities; however, the social science and medical literature contains little research into the perceptions of short-term medical volunteer work from the perspective of members of recipient communities. This exploratory study examines the perception of short-term medical volunteer work in Guatemala among groups of actors affected by or participating in these programs.

**Methods:**

The researchers conducted in-depth, semi-structured interviews with 72 individuals, including Guatemalan healthcare providers and health authorities, foreign medical providers, non-medical personnel working on health projects, and Guatemalan parents of children treated by a short-term volunteer group. Detailed notes and summaries of these interviews were uploaded, coded and annotated using Atlas.ti (Scientific Software Development GmbH, Berlin) to identify recurrent themes from the interviews.

**Results:**

Informants commonly identified a need for increased access to medical services in Guatemala, and many believed that short-term medical volunteers are in a position to offer improved access to medical care in the communities where they serve. Informants most frequently cited appropriate patient selection and attention to payment systems as the best means to avoid creating dependence on foreign aid. The most frequent suggestion to improve short-term medical volunteer work was coordination with and respect for local Guatemalan healthcare providers and their communities, as insufficient understanding of the country's existing healthcare resources and needs may result in perceived harm to the recipient community.

**Conclusion:**

The perceived impact of short-term medical volunteer projects in Guatemala is highly variable and dependent upon the individual project. In this exploratory study, project characteristics were identified that are consistently perceived to be either positive or negative. These findings have direct implications for anyone involved in the planning and execution of short-term medical volunteer projects, including local and foreign medical team members, project planners and coordinators, and health authorities. Most importantly, this preliminary study suggests avenues for future study and evaluation of the impact of short-term medical volunteer programs on local health care services.

## Background

There is growing interest among healthcare providers in the field of global health; over 25% of all 2008 United States (US) medical school graduates participated in global health experiences during medical school. Beyond medical school, there are countless opportunities for physicians to volunteer their services abroad in resource poor countries, frequently in the form of medical missions that last for a week or two at a time. Several editorials in the medical and social sciences literature have raised important questions about potential unintended consequences of such short-term medical volunteer work [1-9]. Editorials such as these raise concern about the ability of short-term volunteers to provide safe and effective medical services in the setting of language and cultural barriers that impair clear communication between patients and healthcare providers. They also raise concerns about a lack of follow-up care for patients who receive treatment from groups with a short-term presence. They raise ethical concerns about people without formal medical training participating in these groups, or medical professionals practicing beyond the scope of their expertise and practice at home, in a setting where they are not held accountable for the consequences of medical interventions made. In addition to basic questions pertaining to patient safety, these editorials raise important questions about the impact of short-term medical missions on the larger medical systems in the countries they visit. For example, it is suggested that short-term medical groups that are not integrated with local medical systems do not understand local medical needs, and consequently, their efforts will be misguided. Furthermore, there is suggestion that groups providing free medical care in other countries undermine the livelihood of medical providers who depend on payment from patients in those countries. The literature in medical anthropology is filled with examples of unintended consequences of medical programs that pay insufficient attention to local conditions and culture and, perhaps more importantly, fail to consider the potentially incompatible and harmful cultural assumptions and values embedded in those programs [10,11]. With countless groups from wealthy countries participating in short-term medical volunteer work abroad, it is critical that we evaluate the safety and effectiveness of these interventions for patients, as well as the larger implications and consequences of such work on the development of medical systems and the health of communities where this work takes place. The editorials summarized above were written by medical professionals from wealthy countries with an interest in global health, and these writings serve as an important starting point in this discussion. Even more important, however, are the opinions and perspectives of those who live and work in the countries where this work takes place, and thus far, their voices have not been heard.

The aim of this study is to expand the critical discussion of short-term medical volunteer work by giving voice to the perceptions of a variety of persons who are involved in, work alongside, or are affected by short-term medical volunteer programs. Because of its geographic proximity to the US and its natural resource base, the US has longstanding political and economic interests in Guatemala. Short-term medical volunteer work may be seen as one extension of those interests in the post-colonial era. As such, short-term medical volunteers often bring with them, albeit unconsciously, attitudes that foster dependence and lack respect for local practitioners and local knowledge and practices related to health. Understanding how short-term medical volunteer work is perceived by those living and working in receiving communities is a critical first step in designing and implementing healthcare programs that provide needed healthcare services to supplement and complement local healthcare systems without undermining their efforts. Specifically, we sought to explore the perceived utility and perceived impact (positive and negative) of short-term medical volunteer work in Guatemala from the perspective of healthcare providers and health authorities in Guatemala. Because of the short time available for the research, this study focuses on the perceptions of these individuals and not on the impact of short-term volunteer programs. Its purpose is to identify and describe the range of perceived issues surrounding short-term medical volunteer work as a basis for future in-depth studies.

We begin with a brief description of the Guatemalan healthcare systems and key health outcomes to provide the reader with an understanding of the context in which short-term medical volunteer programs operate. This is followed by a description of our research methods and findings and a discussion of short-term medical volunteer programs in the context of international aid and development to contextualize the themes identified herein. It is hoped that this report will stimulate further investigation into the specific topics raised within this report.

### Healthcare and Health Outcomes in Guatemala

To understand the perceptions of healthcare providers, healthcare authorities and others working with short-term volunteers in Guatemala, it is important to recognize the provision of healthcare services in Guatemala and health status of the Guatemalan population based on leading health indicators. In 2007, Guatemala's per capita gross domestic product (GDP) was $5,400 US dollars (USD) in purchasing power parity [12], which is 130^th ^out of 228 countries ranked, making Guatemala a "middle income" country on a macroeconomic level. Nevertheless, the income gap between the Guatemalan rich and poor continues to be enormous: 51% of Guatemalans live on less than approximately $2 USD per day and 15% of Guatemalans live on less than approximately $1 USD per day [13]. There is a well established correlation between a nation's income inequality and the health of its population (e.g., infant mortality rate and life expectancy) [14]. In fact, Guatemala is considered to have extreme income inequality among Latin American countries and has the third highest rate of infant mortality and third lowest life expectancy among Latin American countries, behind Haiti and Bolivia [15]. All ethnic groups are affected by poverty in Guatemala (half of Guatemala's 13 million people live in poverty, defined as less than $2 USD per day); however, indigenous Guatemalans, who account for 38% of Guatemala's population, bear a relatively larger burden of the country's poverty. Of Guatemalans living in poverty, 75% (3.7 million people) are indigenous.

The Guatemalan healthcare system is composed of three large sectors: The private sector, an autonomous social security institute, and the public sector. The private sector is subdivided into for-profit and nonprofit healthcare organizations. The for-profit facilities include private hospitals, clinics, pharmacies, and laboratories, all of which essentially offer the full range of services available in most industrialized countries. This sector is typically accessible only to the wealthiest people of Guatemala. As of 2001, less than 5% of the Guatemalan population was covered by private insurance. In 2001, there were approximately 200 nonprofit nongovernmental organizations (NGOs) in Guatemala engaging in health-related activities, 5% of which were estimated to have nation-wide coverage [16]. According to the Swedish International Development Cooperation Agency, there are 90 physicians per 100,000 population (9/10,000) in Guatemala [17], well below the level of 25 physicians per 10,000 population considered adequate by the World Health Organization (WHO) [18].

The Guatemalan Social Security Institute (IGSS) is a formally autonomous institution financed by mandatory contributions from workers and employers based on wages, and it has its own network of services for delivering care. IGSS provides coverage with a limited set of services to formally employed workers, who tend to be urban wage earners. As of 2001, 17% of the population was estimated to be covered by IGSS [16].

The public sector is run by the Ministry of Public Health and Social Welfare (MSPAS). This consists of a network of government hospitals, health centers, and health posts, which are staffed and maintained using public funds. As of 2001, 54% of the population was estimated to be covered by the MSPAS network. According to the PAHO Profile of Guatemalan Healthcare System [16], "the MSPAS does not guarantee the delivery of a package of services, nor do users tend to demand this as a right." As of 2001, 18.8% of Guatemalans were estimated not to have access to any part of the healthcare system described here [16]. Although access to professional medical care is limited to all ethnic groups in Guatemala, it is especially limited to indigenous people [13]. See Table 1 for a summary of key Guatemalan health indices.

**Table 1:** Key health indices-Guatemala [37]

**Life expectancy at birth in 2005**	
Male	65 (years)

Female	71

**Maternal mortality rate in 2000**	240 (per 100000 live births)

**Probability of dying under 5 years of age**	

Overall (2005)	43 (per 1000 live births)

Lowest wealth quintile (1999)	77.6

Highest wealth quintile (1999)	39.3

**Top five causes of death, all ages**	

Chronic obstructive pulmonary disease	53.3 (deaths/100000 population)

HIV/AIDS	49.0

Lower respiratory infection	39.3

Violence	37.1

Perinatal conditions	34.8

It is worth noting that international efforts have been made over the past 40 years to address the inequity in access to healthcare among Guatemalans in the form of numerous development strategies. As an example, in the 1970s, international organizations such as the WHO, the United Nations International Children's Emergency Fund (UNICEF), and the United States Agency for International Development (USAID) financed a program whose goal was to provide rural people with comprehensive primary healthcare services. However, this program was abandoned less than a decade later in Guatemala. It has been suggested that development programs such as these, which filter a great deal of money through the government, are frequently unsuccessful because they often do not address the underlying causes of poverty which are intimately related to poor health outcomes and may even serve to paradoxically reinforce governmental corruption and state suppression of the impoverished communities for which the aid is intended [19].

## Methods

The fieldwork for this paper was conducted in Guatemala between October of 2006 and March of 2007, by two of the authors (TG and HG). Both field investigators were US medical students at the time with advanced but non-fluent Spanish proficiency. Prior to the initiation of fieldwork, the field investigators reviewed qualitative research methods and Guatemalan history and culture. The study was designed in consultation with anthropologists and physicians with prior field experience in Guatemala, and with extensive experience in qualitative research methodology.

In addition to the theoretical reasons mentioned above for choosing Guatemala as the research country, the researchers had multiple local contacts in the study area around the town of Santiago Atitlan. Santiago sits on the southern shore of Lake Atitlan, a large lake in the department of Sololá. The closest facility with higher-level emergency and surgical services is the government hospital in the town of Sololá. Reaching Sololá requires a 30 minute boat ride across the lake, followed by a 30 minute truck or bus ride; the boats do not run after dark. Santiago was historically a regional marketplace where indigenous farmers and merchants from the southern shore of Lake Atitlan and the lowlands to the south of the lake met to buy, sell, and trade goods. Today, it continues to be an almost exclusively indigenous region supported primarily by agriculture and tourism.

To acquire further context and contacts, the field investigators spent their initial 2 months living and volunteering in a small hospital under the supervision of fully trained Guatemalan and US physicians. The hospital in Santiago was established and is funded in large part by a US-based NGO. It is run by a Guatemalan administrative team, and supported by an executive committee made up of both long-term expatriates and Guatemalans living in Santiago. The hospital is staffed by paid Guatemalan physicians and long-term foreign volunteer physicians, as well as a mix of local and foreign volunteer nurses and medical assistants. In addition to its long-term staff, the hospital relies on short-term medical volunteers, including family physicians, emergency physicians, pediatricians, obstetricians and gynecologists, and general surgeons. After the initial orientation phase in the hospital, the field investigators continued to engage in hospital activities, in the spirit of "participant observation." Participant observation, the process of both observing local culture and practices and participating directly in those activities, is an essential component of ethnographic fieldwork where the researcher is her/himself an instrument of data collection [20].

The project was reviewed and approved by institutional review board committees at the University of Colorado Denver in the US and in Guatemala. Over the course of this study, a total of 72 individuals were interviewed. Informants were selected using "purposive sampling," a sampling strategy in which the researchers focus "on selecting information-rich cases whose study will illuminate the questions under study" [21]. This necessarily included a mix of Guatemalans and foreigners. Because the principal aim of this study was to assess Guatemalan perceptions of short-term volunteer work, the Guatemalans we interviewed are considered to be our primary informants, and their statements are most heavily weighted in the Results section of this paper. To understand the perceptions of Guatemalans, we interviewed a total of 23 Guatemalan healthcare providers (seventeen physicians, two nurses, and four community health promoters), five government health officials, and a group of seven parents whose children were treated by short-term medical volunteers. To understand the perceptions of those providing short-term medical services we interviewed 21 foreign medical providers including both short-term volunteers (fourteen) and long-term expatriates (seven), the latter having observed multiple short-term volunteer groups. Finally, we interviewed sixteen non-medical personnel working with a variety of NGOs or health-related projects who, by virtue of their long-term presence in the country, had the opportunity to observe short-term medical volunteers over an extended period and were knowledgeable about the political, economic, and cultural context of Guatemalan health and healthcare. As a group, the respondents varied in their level of interaction with short-term medical volunteers, from extensive to no direct contact. All, however, had knowledge of the presence of short-term medical volunteers in Guatemala and had opinions as to their role in the country.

Interviews were semi-structured, and typically lasted for an hour, although some were significantly longer, and some informants were interviewed on more than one occasion. Most interviews included the two field investigators and a single informant, although we also led two small group interviews. Although we started with an interview guide of questions that we hoped to address with different informants, this guide was used loosely to ensure that information we thought would be significant was included. Following the model of James Spradley [22], these initial, exploratory interviews on a topic that has not been previously addressed in the literature were tailored to the experiences and expertise of our individual informants. Since interviews were not tape recorded and many took place in Spanish followed by our translation into English, many of the quotes are not verbatim, but rather represent closely paraphrased and translated passages that are our best attempt at capturing the idea the interviewee was expressing. All informants were presented with an information sheet in either English or Spanish which explained the goals of the project and provided the informant with written assurance of confidentiality. We quote informants anonymously in this paper to protect their confidentiality and privacy.

One obvious group of people whose perceptions would be important to evaluate are the end-users (i.e. patients treated by short term volunteer groups). However, in designing this study, we elected not to focus on end-user perceptions because it was felt that end-users in the midst of receiving treatment from short-term medical teams would be less likely to offer candid criticism of these groups, especially to two US medical student interviewers. Nevertheless, we did conduct one group interview with seven parents of pediatric patients undergoing surgical treatment by a short-term medical team from the US. In this interview, we asked them why they had pursued care from a foreign medical team rather than through a local medical facility: their comments are briefly addressed in the findings section that follows.

Shortly following each interview, the interviewers created a document summarizing the relevant points made during the interview. Direct quotes captured during the interview by the note taker were also recorded in these interview summaries. At the end of the field research period, these summaries were uploaded into Atlas.ti 5.2 (Scientific Software Development GmbH, Berlin), a computer software program which assists in the analysis of qualitative data. The two field researchers simultaneously reviewed each summary, labeling segments of text with codes that corresponded to the themes (or topics) relevant to the research questions. Once the summaries had been coded and annotated, the interviewers then analyzed all text segments coded under a given theme. These compilations of text segments, coming from multiple interviews but falling under a common theme, served as the basis for each subsection presented in the results section of this paper.

## Results

### Healthcare Needs of Guatemalan Communities

When informants were questioned about what they believed to be the most pressing healthcare needs in Guatemala, a number of public health measures invariably topped the list. The most commonly cited healthcare needs included improved efforts at disease prevention through health education and disease screening programs; improved public health infrastructure; and improved access to primary medical care, particularly in Guatemala's rural areas.

A number of informants focused on poverty as the key determinant of the health disparities between the people of wealthy and poor countries. One Guatemalan surgeon working at a large national hospital stated the problem in the following way:

[Foreign] surgical teams only work on the tip of the iceberg when it comes to addressing the medical problems of this country. The problems of Guatemala – corruption, lack of resources, lack of education – all come from poverty. So poverty is the root of the problem, and surgery does not address poverty.

When the question of healthcare needs in Guatemala was posed to a high-ranking official at the Ministry of Health (MSPAS), he emphasized that the "primary problem in Guatemala is a lack of public health infrastructure and lack of primary care coverage due to a lack of financial resources," further explaining that:

[Short-term medical work] does not, and cannot, address these primary health issues of Guatemala. We already have many surgeons and other physicians who are well trained to take care of all problems common in our country. The lack of healthcare in rural areas is not due to a lack of physicians; it is due to a lack of resources to provide clinics, hospitals, and supplies to these areas.

While none of our informants suggested that short-term volunteer medical work could solve the country's most pressing healthcare needs, there was nevertheless unanimous acknowledgement of the need for increased access to curative medical care, especially for the poorest populations in Guatemala. Informants cited the public healthcare system (MSPAS system), as tending to be the most accessible option to low-income populations in Guatemala. However, a Guatemalan primary care physician working in a foreign-funded hospital explained the pitfalls in the Guatemalan healthcare system:

Even though the national hospitals do not charge anything for their services, preoperative studies are frequently needed for scheduled surgeries. If the national hospital does not have the equipment to do the studies, the patient must go to other places to get them and at times has to pay a lot of money. So even though the national hospital provides health services for free, the patient frequently encounters costs that can prevent a poor patient from receiving necessary treatment.

In addition, given the high levels of poverty discussed above, simply traveling to a healthcare facility can be financially burdensome for a significant portion of the Guatemalan population. Compounding this problem is the paucity of specialists outside of Guatemala City and other larger cities. A physician who is an official at the College of Physicians and Surgeons, stated that "Eighty percent of Guatemala's specialists live and work in Guatemala City, so there is a vast shortage of specialists elsewhere." In explaining the reasons for the lack of Guatemalan specialists working in poor, rural areas, one official at MSPAS stated that:

Physicians working within the public healthcare system are underpaid...the financial incentives to work in a poor area do not exist. All of the specialists end up living in big cities, sometimes splitting their work between public and private practice.

In addition to the economic and geographic barriers to accessing healthcare, language and discrimination were also noted as significant impediments to care. One informant is a Guatemalan employee of a US-funded NGO that works closely with local community leaders in rural villages to seek out patients who are in need of surgery. This organization then coordinates the surgery, linking patients with visiting surgical teams. If needed, they also facilitate and help to pay for the transportation, translators (if the patients do not speak Spanish), accommodations, and food for the patient. This informant reflected that many of the indigenous people (who tend to be those who live in the most rural, poverty stricken areas) are afraid to have surgery and often only speak an indigenous language rather than Spanish, which prevents these patients from entering into Guatemala's public healthcare system. An indigenous Guatemalan whose son was being aided by this US NGO had traveled 8 hours by bus with her son who was awaiting hand surgery from a US short-term surgical team. She stated that she felt physicians at the national hospitals helped those with money first, and then, if there is time, they would see the poor last.

### Dependence on Foreign Providers

Over the course of our interviews, the issue of dependence was frequently raised by both Guatemalans and foreigners. One repeatedly cited criticism was that foreign medical projects remove or lessen the incentive for the government to invest in healthcare for their own people. A Guatemalan physician who works in a foreign-funded hospital which is currently the only hospital in the area offering 24-hour emergency and surgical/obstetrical care is, along with a number of other physicians in the area, petitioning the government to build a full-service, government-run health center in his area. He explained that in deciding where to invest money in improving healthcare services, the government "only considers the number of existing healthcare services already in the area, regardless of the quality of services provided." Thus, the presence of multiple NGO health projects in the area may actually impede development of the area's public healthcare infrastructure.

In addition to the potential for governmental dependence on foreign medical aid, many informants described the problem of patient reliance on free medical and/or surgical care provided by short-term volunteers. A Guatemalan administrator working in a local NGO which provides reproductive health services throughout Guatemala, expressed her concerns regarding free care provided by foreign medical groups:

Patients get used to the free care and end up waiting for the next group to arrive to give them free care rather than seeking out ways in which they can help themselves. What will happen when all the NGOs leave? The people won't know how to go about finding a way to get care.

Similar sentiments were noted by an American surgeon and head of an NGO in Guatemala, who stated, "If a volunteer group provides free healthcare, the community can become spoiled and end up relying on that service rather than on the permanent [government-run] system which already exists."

### Patient Selection and Payment Systems

When our informants were questioned about ways in which dependence on foreign aid could potentially be avoided, appropriate patient selection and attention to the payment system were most frequently mentioned. When discussing the issue of patient selection, there was almost universal agreement between both Guatemalans and foreigners that short-term volunteer groups should focus their services on the populations who are most in-need. The most frequently cited challenge to shot-term medical volunteer work was the task of reaching the patients who truly cannot afford other options for medical attention. We spoke with a Guatemalan physician working in a clinic that was hosting a North American short-term surgical group. He expressed his concern that the aid provided by volunteers may not actually be reaching the poorest people in Guatemala and emphasized that if patients who can afford to pay for their own private care receive free care from foreign volunteer groups, those volunteer groups end up competing with the private Guatemalan physicians (who could perform the same surgeries, but for a fee) for patients. He went on to describe the challenge of trying to suggest to the North American group that they perform a financial evaluation of all patients in order to help target those who truly cannot afford to pay for surgery. He stated that he sensed that the North Americans "seem to perceive everyone in Guatemala to be poor, and therefore do not think it is important to do a socioeconomic evaluation."

Informants' opinions on which payment system should be used by short-term medical groups were varied. One head coordinator of a short-term medical volunteer group stated that their group provided "completely free surgical care to every patient without an evaluation of their ability to pay." A number of informants criticized this form of care, suggesting that it becomes "detrimental to society" by causing disinvestment in healthcare by the government to take care of their own population, dependence on outside aid, and competition with the existing healthcare system.

A few informants were of the opinion that short-term medical volunteer work should be free to those patients who cannot afford care in Guatemala. One foreign-born surgeon, who has been operating full-time in poor countries for nearly 20 years, stated that he provides completely free surgery to the "poorest of the poor" through a private foundation. He described why he chooses to do this in the following way:

Last year, I did over 5000 free surgeries for the poor around the world and if my patients would have had to pay for this care, I probably would have done half that number of surgeries. The poorest patients do not have the resources even to be able to afford the transportation, accommodation and food while they are in the hospital, let alone the surgical and medical care...What's the definition of charity if it's not free?

In addition, two out of the four health promoters working in rural, poverty-stricken areas described the free care provided by short-term medical volunteers as one of the greatest benefits to their patients. One health promoter stated, "If [patients] have to pay for their care, some are so poor that they will have to choose between paying for food and paying for their medical care."

Of the 20 informants who discussed the issue of payment directly, fourteen believed that all patients should pay something for their treatment. Most believed that when patients were asked to pay for their treatment, they were in a better position to feel as though they had ownership of their own care, rather than being passive informants in that care. A leader of a US NGO that seeks out patients in rural areas in need of surgical care always has the patient pay something for this service (often it is only a few quetzals – equivalent to less than $1 USD). He described his reasoning in the following way:

I remember talking to a couple of patients who came back from a free surgical [short-term medical volunteer group] who were dissatisfied with their care. When pressed for why they were dissatisfied, they said the facility made them clean up their own area, or they didn't have tortillas – small, irrelevant reasons for their dissatisfaction with their care. I have never had that experience with patients who have to pay something for their care.

Another administrator at a Guatemalan NGO echoed these sentiments by saying, "Even the poorest people in the country can find five quetzals. The point isn't to cover the cost of the care. Rather, the point is to get people to take more responsibility for their own care."

Nearly all of the informants who believed in asking for payment from patients (including Guatemalan healthcare providers, health authorities, community members, and foreigners) suggested using a sliding scale system of payment, in which the amount patients are asked to pay is based on a careful socioeconomic screen performed by social workers and/or leaders of the patient's community, who are in the best position to know what the patient can actually afford to pay. Again, the informants emphasized that the payments should never jeopardize the patients' ability to obtain health care.

### Burden on Host Organization/Community

Another major theme frequently discussed by the informants was that short-term medical volunteers have the potential to be quite burdensome (both financially and in terms of personnel time) for host organizations and communities in Guatemala. It should be noted that nearly every Guatemalan interviewee expressed appreciation for the service that visiting teams provided to their communities and many acknowledged the personal sacrifices that individual volunteers made in order to provide these services. Nevertheless, there was also a great deal of discussion about how this type of work can become financially burdensome for the host organization. One Guatemalan project coordinator of short-term medical volunteers expressed that he felt he was "half project coordinator and half tour guide. I have to arrange transportation, accommodation, food, and translators for all of the volunteers."

Many informants noted that a big disadvantage to short-term medical volunteer work is the strain on local personnel time when the volunteers did not know the language or were unfamiliar with the clinic setting. A project coordinator of a US NGO stated that, "When the volunteer doesn't speak the language, misunderstandings can occur and cause big problems, not only for patients, but also for local staff who work with the volunteers."

Some short-term medical volunteer organizations have tried to combat this problem by asking their volunteers to pay for their own expenses. The head of an NGO which regularly organizes surgical short-term medical volunteer work in a private hospital in Guatemala, expressed the following thoughts:

We get into trouble when physicians just bring their hands. We ask all of our volunteers to cover their own expenses, such as travel, lodging, and food. We also cover the cost of each surgery, including supplies and electricity in the operating rooms, and to offset the financial burden on the hospital of providing follow-up care, our visiting groups make a donation to the hospital for each patient they operate on. We understand that it is very expensive for any facility to host short-term volunteers.

Numerous informants suggested that it is best to limit the number of people on a visiting medical team to only those who are necessary, as large groups tend to get in the way of the regular operations at host facilities and end up being a rather large burden. As an extreme example, a physician who has worked on various medical aid projects around the world, told us of a visiting medical team from the US which brought 78 people, including surgeons, primary care physicians, nurses, cooks and translators. He continued:

Guatemala already has doctors, nurses, cooks and translators. So, it would be better to bring the specialists that may be needed and then utilize as many in-country personnel as possible to carry out the mission. In that way, you are wasting less money, strengthening the country's healthcare resources, helping the country's economy, and increasing the quality of care.

### Coordination

Many Guatemalan informants talked about a level of arrogance or elitism that they often see in visiting medical professionals. Most of these informants noted that when foreign providers work in coordination with the local healthcare providers, it reflects an acknowledgement that the local providers are competent. Working in isolation from the surrounding medical community was perceived to reflect the opposite sentiment. Furthermore, the respect shown to local providers by working alongside them is also perceived to be visible by the local patient population, which has a positive impact on the local provider's relationship with their community.

Some Guatemalan physicians described their frustration with visiting medical teams who work in isolation from the local medical community. A Guatemalan surgeon who works in a private clinic as well as a national hospital poignantly stated:

Guatemalan patients, especially those with less education, tend to put more faith in a blonde haired, blue eyed, white skinned foreign physician than their own Guatemalan physicians. These foreigners show up with their shiny new equipment and do their free surgeries without ever working with any of [the Guatemalan physicians]. US doctors come to Guatemala and practice medicine when and where they want. Guatemalan doctors may have a hard time even entering the US, let alone being able to practice medicine there. US physicians are not superior to Guatemalans. I am perfectly capable of taking care of my own people.

In discussing the utility of short-term medical volunteer work with the co-founder of a successful NGO that organizes US surgical teams to perform surgeries in Guatemala, he said, "Short-term volunteer work can be completely effective if it's attached to a long-term program." The importance of short-term medical volunteers coordinating their activities with groups that have a long-term presence in Guatemala was by far the most frequent recommendation made by our informants. In fact, it was often more of a demand than a recommendation, with some informants commenting that short-term medical volunteer work that is not coordinated with a long-term presence is "the worst kind of care," or that those short-term medical volunteers "might as well stay home."

When describing the benefits of coordination, one long-term foreign volunteer noted that the local healthcare provider could offer the short-term medical volunteer knowledge of resources, customs, and opportunities available to the local population. In addition, by coordinating with a long-term presence well in advance, many informants pointed out that the local contact is able to recruit patients for the volunteer group to see.

Additionally, coordination with a local, long-term presence is a legal requirement in Guatemala. In order for visiting healthcare providers to practice medicine in Guatemala, they are required to register with the College of Physicians and Surgeons (*Colegio de Médicos y Cirujanos*), providing evidence of credentials and a Guatemalan physician contact. Nevertheless, a number of Guatemalan health authorities and healthcare providers expressed concern that many groups of foreigners practice medicine in Guatemala without communication or coordination with the local healthcare system.

### Meeting the Needs of the Community

Groups that do not work in coordination with a long-term presence frequently provide services that do not match the needs of the community. Many informants talked about "de-worming campaigns" in areas without clean drinking water sources; groups that provided free eye glasses without an eye exam; or groups that indiscriminately handed out vitamins as examples of particularly misguided interventions which reflect the lack of coordination and consultation with the local healthcare community.

Another detrimental effect of groups who practice in isolation is that services already provided by the Guatemalan community end up being duplicated by the volunteers. For example, we spoke with a Guatemalan physician working at a government health post in a community that was recently devastated by a natural disaster. His area regularly receives many foreign medical aid groups; however, "very few have actually come [to his health post] to ask about what is needed." He further described the problems with this lack of communication, citing an example of a short-term medical volunteer group who saw patients over a weekend and provided medications without any records or understandable explanations to the patients of why they needed the medication. He said those same patients came to his health post the following week, unable to explain what was done and why they were taking medication, forcing him to repeat their exams without any benefit to the patient or the system.

Many informants pointed out that at the very least, it is important to be in contact with local providers to ensure that what the volunteers are doing is actually needed and desired in the community. As one long-term foreign volunteer stated, "First understand if the people who you plan to help actually want it."

### Follow-Up Care

Follow-up care frequently came up in the context of why coordination with a long-term presence is important. As one interviewee pointed out, "Most problems take longer than one week to fix – without continuity, the care is not complete." In addition, many Guatemalan healthcare providers expressed willingness to provide the follow-up care to patients with whom they had personal contact, but stated that providing follow-up care to patients with whom they were unfamiliar could be problematic. Many informants suggested that one way to minimize incomplete care in the surgical field is to provide a record of what was done and why (in the appropriate language) to each patient, to the facility in which the surgery took place, and to the physician who will be responsible for the follow-up care.

One nonprofit private hospital was often cited as being particularly excellent at providing follow-up care. This hospital, through a small number of international NGOs with which they coordinate, hosted surgical teams from North America and Europe year-round and provided very low-cost surgeries to pre-screened patients who were in need. They involved Guatemalan surgeons and support staff in the surgery, and had patients return to that same hospital (where each patient's records were kept) for their follow-up care. They also hired a Guatemalan surgeon whose primary responsibility was to take care of post-operative patients and complications which arose from surgeries performed by foreign volunteers.

### Resource and Information Sharing

Surprisingly, a number of the foreign volunteers were quick to point out that the benefits of short-term medical volunteer work may be greatest for the volunteers themselves. However, the majority of our Guatemalan informants (including healthcare providers, health authorities, and Guatemalans working on other health projects) as well as the long-term foreign volunteers also emphasized the fact that if coordination exists between visiting and local healthcare providers, these short-term medical interventions can be a positive experience for the local providers as well. Many Guatemalan informants described the educational opportunities for both sides when visiting teams work together with the Guatemalan providers. Others suggested educational exchanges between US and Guatemalan medical schools and sending the Guatemalan physicians to educational conferences as ways to provide mutually beneficial interactions.

The Guatemalan informants often cited the donation of equipment, medications, and supplies as one of the greatest benefits of short-term medical volunteer work. A Guatemalan ophthalmologist in private practice pointed out that:

There is a cost for the local ophthalmologist to provide follow-up care to patients who cannot pay for it, so there needs to be a reciprocal benefit to the relationship. Money is not the solution – that disappears and doesn't get to the patients. But, if volunteers leave something behind for the local physician, such as equipment, medications, operative instruments, or supplies that the physician could continue using when the volunteer group leaves, that benefits us *and *our patients.

It was often stated that donations amplify the impact of short-term medical volunteer work, as they improve the quality of services offered even after the volunteers are no longer present. However, the recipients of these donations often talked about the vast amount of expired medications they receive, which amount to what one interviewee referred to as "trash" that must be sorted through and disposed of, thus wasting valuable staff time. The argument that expired medications were "better than nothing" was not supported by our informants, as one interviewee commented, "If the medications aren't fit for human consumption in the US, why should they be fit for human consumption in a poor country?"

### Quality of Care

Many of the foreign volunteers and volunteer coordinators focused on the issue of quality of care when practicing outside of one's own country. They talked about striving to provide the same quality of care as one would at home and working first and foremost out of responsibility and respect for the patient. As one long-term volunteer put it, "Always keep in mind that you are there to provide the best possible care for the patient – do things because the patient needs them, not for your own experience." They emphasized using good judgment in making medical decisions, including conservative patient selection for surgical cases. Many volunteers also discussed the importance of knowing your limits as a visiting physician and restricting your work to cases that are within one's technical limits and that fit the resources of the setting.

Along the lines of professional judgment, many informants (both Guatemalan and foreign) expressed concerns that some short-term medical volunteer groups may be trying to see too many patients per day at the expense of quality of care to the patients. Informants often worried that when volunteers focused on the number of patients seen per day, rates of complications increased, misdiagnoses and inappropriate treatment abounded, and patient education plummeted. In addition, the majority of our informants believed that religious and political discussion should be kept separate from the provision of patient care.

## Discussion

Our study, although small in scope, is one of the first to systematically and critically examine the effects of short-term medical volunteer work. All major thematic areas in our results underline the challenges of outside groups working as equal partners. Is it paternalism or cooperation? Is it charity or aid? Is it experimentation or quality care? Have all stakeholders been properly identified? Let us say that a recipient community has been appropriately consulted and involved to develop the most suitable intervention with strong community ownership. Omitting other healthcare providers, organizations, and the Ministry of Health may nevertheless jeopardize the long-term success and sustainability of any effort. The very real power and wealth differential between short-term medical groups and their host communities make trust, understanding, and true partnership difficult.

The complex nature of feelings toward short-term medical volunteer groups in our study parallel the often nuanced and contradictory feelings toward the US and industrialized countries. Guatemalans have particular reason to be suspicious of the US: The US-based United Fruit Company and the Central Intelligence Agency coordinated the overthrow of democratically elected Guatemalan president Jacobo Arbenz in the early 1950s and brought an end to important social progress in Guatemala [23,24]. This US-backed "regime change" ushered in a 40 year period of state-sponsored terror, which resulted in up to 200,000 deaths and disappearances, and the displacement of over 1 million Guatemalan people [24-26].

The challenges facing foreign providers do not negate the potential benefits of external assistance. Despite its ranking as a middle income country, Guatemala "holds some of the poorest health records in the Americas" and "holds the third-lowest position in the Americas in percentage of GDP dedicated to both private and public health care – 4.4%" [27]. Because of widespread poverty in rural areas and poor compensation for physicians in the public healthcare system, there is little incentive for Guatemalan physicians to work in poor communities. Cuba's medical assistance program helps bridge the gap, in a manner that is directly integrated into Guatemala's healthcare system. In 2002, 514 Cuban doctors were working in rural areas of Guatemala to staff public health clinics run by the Sistema Integral de Atencion de Salud (SIAS), the national health care system established under President Alvaro Arzu (1996–2000) that increased coverage in rural areas by 90% [27]. Cuban healthcare providers often stay for two years or more, have language on their side, and lack some of the baggage of health professionals from the US.

Short-term volunteer groups may yet identify a framework to contribute meaningfully. Very few have attempted to identify guidelines that would make short-term medical work more effective, despite its limitations. Suchdev et al listed seven guiding principles from their experience operating short-term medical groups out of the University of Washington: A clearly defined mission, close collaboration with the recipient community and its institutions, a focus on sustainability, education for the short-term team and the community, service by addressing true health needs, teamwork among short-term volunteers, and rigorous program evaluation [28]. Much of the remainder of the existing literature rightfully draws attention to the pitfalls of short-term volunteer work, but has little to offer in terms of a generalizable solution [1-7]. To date, it appears that more scholarly attention has been directed to the educational, ethical and practical issues facing medical students on international electives [29-33]. The situation of these medical students is similar in many ways to that of short-term medical volunteers; they are often poorly prepared, poorly connected, and tempted to practice outside of their scope of competencies. Those unprepared will repeat the same mistakes, as for example, in Paul Farmer's words, "conflate poverty with culture" [34]: Attributing differences in healthcare practices and decisions to different cultural beliefs, rather than to lack of resources and basic services.

According to our results, recipient communities may perceive very tangible benefits from short-term volunteer groups: Free or discounted care, improved access to healthcare overall, access to highly-trained specialists, and access to procedures not always possible within the local infrastructure. Local providers enjoy exchanging experiences and knowledge with foreign visitors, and appreciate the influx of supplies that accompany volunteer groups. On the negative side, it appears some of the least sophisticated groups offer services or treatment that are seen to be at best duplicative, and at worst, harmful. For example, though some drugs may remain effective 1–2 years past their expiration date, the perception of harm may arise from using drugs that are no longer considered safe, legal, or effective in the US. Similarly, a surgical group not planning for appropriate local follow-up could also be seen as acting recklessly and creating the potential for harm. Such issues may be easily solved with proper planning and supplies. On the other hand, many situations described by our respondents do not present the opportunity for an easy fix. Well-intentioned, well-prepared groups provide services that may help many but may harm others though unforeseen externalities. For example, free care from outsiders improves access in the short-run, but may undermine local healthcare providers, and in the long-run may reduce access: The government might close public clinics with patient volumes that are dropping, and private physicians might leave for areas without competitors providing free care. This could only further increase the dependence on external assistance. Significant externalities in medical assistance are not unique to short-term medical volunteer groups. Garrett describes "Dutch Disease" in developing countries, where large expertly-planned and externally-funded vertical health programs draw human and material resources away from primary care, and from other vital sectors of the economy [35].

The debate over free care, raised by some of our respondents, has a long history and continues vigorously in the development circles [36,37]. Some believe that patients and communities will only truly take ownership of and responsibility for their healthcare if they have to pay for it. Other groups, such as Partners in Health  argue that healthcare is a right and that any fee is a barrier to health care access for the poor [34].

Our study has several limitations: 1) It relies on targeted expert informants rather than on the direct recipients of medical care from short-term medical volunteers; 2) It examines only one small part of the world, thus making generalizations difficult; 3) It lacks external reference points, given the scarcity of research on short-term medical work mentioned; and 4) It used field investigators who were outsiders: American medical students who were non-native Spanish speakers. Though there is little formal research with which to compare our results, the issues raised by authors previously cited [1-7] are similar across continents and countries, thus mitigating limitations 2 and 3. Regarding limitation 4, it would appear from their responses that many informants had no difficulty being frank and open with the field investigators. Language barriers may indeed have impeded understanding of subtle nuances; however, the concordance of responses, as well as their complexity, would suggest, at a minimum, adequate comprehension.

The possibilities for future research in short-term medical work abound: With regard to the perceptions of Guatemalan healthcare providers and authorities to short-term volunteer work, a more in-depth analysis of how informants' place in the Guatemalan healthcare system and in the global political economy of healthcare (e.g., links to foreign medical schools, to local, regional, and international NGOs, national and international professional associations, and religious organizations) would further illuminate the complex gradients of dependence and the flow of resources. With regard to short-term medical volunteers, surveys and group and individual interviews could assess their attitudes toward and perceptions of the countries in which they have worked both before and after service to improve preparation for volunteer work and design programs that build more truly reciprocal relationships. With regard to recipients of healthcare from short-term medical volunteer groups, future studies should seek to understand who pursues care from short-term medical volunteers, why, and under what circumstances. Are there perceived or actual differences in quality or type of care? Are patients and their families satisfied with their care? Does seeking and obtaining care from foreign providers carry different social meanings, e.g., greater status, than care from Guatemalan providers or state-run clinics? Finally, it is our hope that this paper will stimulate studies into the economic, political, and health outcomes of short-term volunteer programs to critically assess their quality and effectiveness. What is the effect of the concentration of such services on the government investment in healthcare infrastructure and services in those areas? Do free or very low cost services provided by short-term volunteers truly draw patients away from private practitioners or state services? Are outcomes for procedures (e.g., cataract removal) or conditions (e.g., diabetes) different when care is provided by the regular healthcare system versus by short-term medical volunteers?

## Conclusion

The perceived impact of short-term medical volunteer projects in Guatemala is highly variable and dependent upon the individual project and the perspective of the observer. In this exploratory study, certain project effects were repeatedly identified as being either positive, such as improved access for the underserved, or negative, such as drain on local resources. Other responses highlighted the complex consequences of short-term medical volunteer work, through unforeseen externalities on the healthcare system. These findings have direct implications for anyone involved in the planning and execution of short-term medical volunteer projects, including local and foreign medical team members, project planners and coordinators, and health authorities. Most importantly, this study suggests avenues for future study and evaluation of the impact of short-term medical volunteer programs on local health care services.

## Competing interests

The authors declare that they have no competing interests.

## Authors' contributions

HG and TG carried out all interviews and data analysis and interpretation. All authors contributed to the conception and design of the study, all authors were involved with drafting and critical revisions of the manuscript, and all authors read and approved the final manuscript.
